# Lower incidence of respiratory infections among iron-deficient children in Kilimanjaro, Tanzania

**DOI:** 10.1093/emph/eox010

**Published:** 2017-06-28

**Authors:** Katherine Wander, Bettina Shell-Duncan, Eleanor Brindle

**Affiliations:** 1 Department of Anthropology, Binghamton University (SUNY), Binghamton, NY, USA; 2 Department of Anthropology, University of Washington, Seattle, WA, USA; 3 Center for Studies in Demography and Ecology, University of Washington, Seattle, WA, USA

**Keywords:** nutritional adaptation, nutritional immunity, optimal iron hypothesis, evolutionary epidemiology

## Abstract

**Objective**: We posited a trade-off in iron nutrition, with iron deficiency decreasing risk for infection by depriving infectious agents of iron while increasing risk for infection by compromising immune protection. We described associations between iron deficiency and prevalent and incident infectious disease episodes and cell-mediated immunity (CMI) among 283 children in Kilimanjaro, Tanzania.

**Methodology**: Whole blood specimens were evaluated for hemoglobin and dried blood spots (DBS) were evaluated for biomarkers of iron deficiency (transferrin receptor) and inflammation (C-reactive protein and α_1_-acid glycoprotein). Prevalent and incident infectious disease episodes were identified by physician’s diagnosis. CMI was evaluated as delayed-type hypersensitivity to *Candida albicans* (DTH-*Candida*). Associations between iron status and elevated inflammation, prevalent infectious disease episodes and DTH-*Candida* were described with logistic regression models; associations between iron status and incident infectious disease episodes were described with Cox proportional hazards models.

**Results:** Elevated inflammation and diagnosed infectious diseases were more common among children with iron-deficiency anemia (IDA, severe iron deficiency), but not significantly so. The incidence of infectious disease was lowest among children with moderate iron deficiency (iron-deficient erythropoiesis, IDE); this pattern was most apparent for respiratory infections (aHR: 0.24; p: 0.030). DTH-*Candida* was not compromised among children with any degree of iron deficiency.

**Conclusions and implications:** We observed no adverse effect of iron deficiency on CMI, but did observe patterns consistent with the hypothesis that moderate iron deficiency *protects against* respiratory infections and may represent a nutritional adaptation to infectious disease. This suggests that interventions targeting iron deficiency should be coupled with effective infectious disease control measures.

## Teaser

While unhealthy, iron deficiency may be adaptive, by preventing respiratory infections in environments like Kilimanjaro, Tanzania, where infections are common. It is important that programs to prevent iron deficiency be partnered with programs to prevent infectious disease transmission to improve children's overall health.

## BACKGROUND AND OBJECTIVES

Iron is an essential micronutrient for child health; it is employed in cellular respiration and oxygen transport. Iron deficiency—iron availability that is inadequate to meet tissue iron needs—and anemia—inadequate hemoglobin—compromise children’s growth, cognitive development and motor skills development [1]. Iron deficiency also seems to limit the function of immune cells and thus may increase children’s risk for infectious disease [1–3]. Iron deficiency is one of the most common forms of malnutrition world wide, and has been targeted for public health action by the World Health Organization [4]. However, iron deficiency may also protect against infectious disease by denying infectious agents iron [5, 6]. This may create a trade-off in dietary iron intake—more iron available to immune cells *vs.* more iron available to infectious agents—such that optimal protection against infectious disease occurs at a level of dietary iron intake and availability that is inadequate to meet tissue iron needs. In other words, dietary iron deficiency may be optimal for infectious disease risk; even if unhealthy, iron deficiency may constitute a *nutritional adaptation* to infectious disease.

Iron is an essential nutrient for disease-causing infectious agents, and the object of an on-going evolutionary ‘arms race’ [7]. Infectious agents have evolved multiple mechanisms to access host iron, such as the diverse and powerful array of siderophores—iron binding and uptake compounds—produced by bacteria [7–9]. Mammals have evolved multiple mechanisms to limit infectious agents’ access to iron: iron chaperone proteins, such as transferrin and lactoferrin, bind iron with high affinity; macrophages sequester iron and intestinal tissues limit iron absorption during an inflammatory response to infection; and, immune cells produce siderocalin, a protein that interferes with the action of siderophores [7–9]. Infectious agents have, in turn, evolved multiple mechanisms to subvert and exploit these defense mechanisms: many bacteria and protozoa express receptors for human iron-binding proteins (heme, hemoglobin or transferrin); some bacteria and protozoa have the ability to exploit the iron-rich niche of iron-sequestering macrophages; and, diverse bacteria produce ‘stealth’ siderophores which are not bound by siderocalin [10–12]. To the extent that humans are losing the arms race over iron [7], dietary iron deficiency may be a particularly effective means to limit infectious agents’ access to host iron, and thus may constitute a nutritional adaptation to infectious disease.

Prospective studies in Kenya, Tanzania and Malawi have shown clearly lower rates of placental malaria among iron deficient pregnant women [13, 14] and lower malaria incidence, severity and mortality among iron deficient children [15–17]. Iron deficiency may also protect against tuberculosis [9]. Whether these observations generalize to other infectious diseases or infectious disease as a whole is unclear, however, as iron deficiency may have different, even opposite, effects on risk for infection across infectious agents. A systematic review of studies conducted primarily in European healthcare settings demonstrated no effect or increased risk for incident infections among those with iron deficiency, particularly iron deficiency anemia (IDA) [18]; however, the generalizability of research conducted in healthcare settings in the western world is also limited.

To date, two studies have investigated the hypothesis that iron deficiency may protect against infectious diseases in general among children in East Africa. Both employed biomarkers of inflammation to identify putative prevalent infectious disease episodes, as well as biomarkers of iron status, and both examined cross-sectional associations between iron status and elevated inflammation among children. These studies document opposite patterns in Tanzania and Kenya, and provide limited support for the hypothesis that iron deficiency protects against infectious disease. Among 5–10-year-old children in northern Kenya, the prevalence of infectious disease was highest among iron replete children (adjusted odds ratio, aOR: 2.9; *P* = 0.01) and no evidence of increased infectious disease prevalence among children with IDA due to compromised immune protection was apparent [6]. However, among a nationally representative sample of 6–59-month-old children in Tanzania, the prevalence of infectious disease was highest among children with IDA (aOR: 2.6; 95% CI: 1.76, 3.70) [19]. The disparity between these two studies, which employed very similar biomarkers and analyses, may be attributable to differences in the infectious disease ecologies of northern Kenya and Tanzania. They may also reflect reverse causation in the cross-sectional sample from Tanzania, as infectious diseases may be able to *cause* iron deficiency through many routes, including blood loss (e.g. hookworm) [20], destruction of red blood cells (e.g. malaria) [21], direct interference with iron absorption in the stomach and gut (e.g. *Helicobacter pylori* and *Giardia spp*) [22, 23] and prolonged inflammation [9, 12].

Here, we investigate associations between iron status and both prevalent *and incident* infectious diseases, as well as CMI, among children in Kilimanjaro, Tanzania. Inclusion of incident infectious diseases in these analyses is important because incident infections are *new* infections, occurring *after* iron status was characterized. Thus, these analyses are much less vulnerable to reverse causation than are cross-sectional evaluations of associations between iron status and prevalent infectious disease. We tested the predictions that (i) the prevalence of infectious disease will be lower among children with iron deficiency (particularly mild-to-moderate iron deficiency) than among iron replete children; (ii) the incidence of infectious disease will be lower among children with iron deficiency (particularly mild-to-moderate iron deficiency) than among iron replete children and (iii) intact CMI will be more common among iron replete children than among children with iron deficiency (particularly severe iron deficiency).

## METHODOLOGY

Study population and sampling

We performed secondary analysis of specimens and data originally collected to evaluate associations between early exposure to infectious agents and cell-mediated immunity (CMI) [24, 25] among children in Kilimanjaro, Tanzania. Data and specimens were collected in the spring of 2010, by the first author and four field assistants (local residents and medical personnel trained in data collection techniques) in a facility belonging to Nshara Community Medical Centre (NCMC). Procedures were approved by the Institutional Review Board of the University of Washington (UW) and Tanzania’s National Institute for Medical Research. Parents of participating children provided written informed consent.

A census of all 2–7-year-old children in the study area was made; 400 were randomly selected and invited to participate, for a target sample of ∼300. All HIV-negative children living with at least one parent and living in the study area for at least the preceding 6 months were eligible to participate. All participating 3–5-year-old children were invited to enroll in active surveillance for infectious disease signs/symptoms, with a target sub-sample of ∼50.

### Study design

Participating children were administered mebendazole, an anti-helminthic medication, prior to participation to eliminate any prevalent helminth infections, which could have affected both iron status and immune outcomes. Children’s iron status, CMI, inflammation and prevalent infectious diseases were characterized upon initial participation in data collection. Passive surveillance for incident infectious diseases was conducted for the remainder of the study for all children, and active surveillance was conducted for the 3–5-year-old sub-sample.

Iron deficiency was characterized with the biomarker transferrin receptor (TfR). Transferrin is an iron ‘chaperone’ molecule. Cells acquire iron bound to transferrin via TfR. Cells’ expression of TfR increases as tissues experience iron stress, thus increases in soluble TfR can be used to identify iron deficiency [26]. Importantly, TfR is more robust to inflammation than other commonly used biomarkers of iron deficiency, such as ferritin.

Anemia (inadequate hemoglobin) was characterized directly (with blood hemoglobin).

Cell-mediated immunity was characterized with an *in vivo* challenge of delayed-type hypersensitivity (DTH) [24, 25]. DTH testing introduces a small amount of the surface antigen of an infectious agent (in this case, *Candida albicans*, a ubiquitous fungal pathogen) under the most superficial layers of the skin. If CMI is intact, evidence of a memory immune response is apparent after ∼24 h as an induration at the test site. Children with negative DTH have higher risk for infectious diseases [27].

Inflammation was characterized with two widely used biomarkers of inflammation, C-reactive protein (CRP) and α_1_-acid glycoprotein (AGP) [28]. Clinically apparent infectious diseases were diagnosed by physicians both at the time of initial data collection (prevalent infections) and subsequent to initial data collection (incident infections), and the sub-sample of 3–5-year-old children participating in active monitoring were visited weekly in their homes and any signs/symptoms of infectious disease in the preceding week were recorded.

### Data collection

Five hundred milligrams of mebendazole was administered upon recruitment (1–7 days prior to data collection), unless parents reported a child had not previously received mebendazole. Primary caregiving parents or relatives completed a questionnaire regarding the child’s family and household composition, household environment and medical history. Caregivers were asked to report any signs/symptoms of illness (open-ended) in the preceding 2 weeks, and specifically cough, sneeze, runny nose, sore throat, fever, diarrhea, vomiting, stomachache, headache, weakness/fatigue, as well as any medical care sought and diagnoses and treatment given.

A sterile lancet was used to obtain capillary whole blood by finger-stick. Capillary blood was used to screen for HIV with a rapid diagnostic test (SD BioLine HIV-1/2 3.0 rapid HIV-1/2 test) and HIV positive children were excluded. Hemoglobin concentration was estimated from capillary blood with a hemoglobinometer (HemoCue Hb 201^+^). Additional capillary blood was allowed to fall freely onto filter paper (Whatman #903 Protein Saver Cards) for dried blood spots (DBS). DBS were allowed to dry for < 24 h, frozen in the field, and shipped on dry ice to the Biological Anthropology and Biodemography Laboratory at the University of Washington, where they were stored at −20°C until assay.

Weight was measured with a digital scale (Tanita) and height with an anthropometer (GPM). Triceps skinfold (TSF) thickness was measured with a Lange Skinfold Measurement Caliper (Graham-Field). Temperature was measured with an aural thermometer (Braun Thermoscan).

The Candin (Allermed Laboratories) skin test for delayed-type hypersensitivity to *Candida albicans* antigen (DTH-*Candida*) was administered by introducing 0.1 ml of challenge antigen under the most superficial layers of the skin of each child’s forearm with a 27-gauge tuberculin syringe. Approximately 24 h after administration, the site of the test was evaluated for the presence of an induration, and the size of any apparent induration was measured in millimeters in two perpendicular diameters.

To characterize prevalent infectious diseases, children judged by field assistants to be ill at the time of data collection were referred to NCMC, and diagnoses given by NCMC physicians were recorded. For passive surveillance for incident ID, caregivers of children in the entire sample were encouraged to return for referral to NCMC if they perceived in a participating child any need for medical care subsequent to the initial data collection visit, and diagnoses given by NCMC physicians were recorded. Active surveillance for 3–5-year-olds for signs/symptoms of ID consisted of weekly home visits. Caregivers were asked to report any signs/symptoms of illness in the time since the initial data collection or preceding home visit, and specifically to report cough, sneeze, runny nose, sore throat, fever, diarrhea, vomiting, stomachache, headache, weakness/fatigue, as well as any medical care sought and diagnoses and treatment given.

### Laboratory analysis

DBS specimens were analyzed in the Biological Anthropology and Biodemography Laboratory for transferrin receptor (TfR), C-reactive protein (CRP) and α_1_-acid glycoprotein (AGP). TfR [29] and AGP [28] were assessed with kit assays modified for use with DBS; CRP was assessed with an in-house assay [30]. Intra-assay coefficients of variation (CVs; for the assay runs included in this analysis) were: TfR, 8.1%; CRP, 16.2%; AGP, 10.6%. Inter-assay CVs were: TfR, 7.3% at low and 3.5% at high concentration; CRP, 11.9% at low and 10.2% at high concentration; AGP, 18.6% at low and 9.8% at high concentration.

### Data analysis

Iron deficiency was defined as TfR > 5 mg/l [6]. Anemia was defined as Hb < 11 g/dl for children < 5 years and Hb < 11.5 g/dl for children ≥ 5 years [31]. Iron status was defined as: *iron replete:* no iron deficiency, no anemia; *iron-deficient erythropoiesis (IDE):* iron deficiency, no anemia; *iron deficiency anemia (IDA):* iron deficiency, anemia; *non-iron deficiency anemia (NIDA):* no iron deficiency, anemia.

*Prevalent infectious diseases* were those identified in children upon their initial participation in data collection by reported signs/symptoms or referral diagnosis. *Incident infectious diseases* were those identified subsequent to children’s initial participation in data collection by reported sign/symptoms (active surveillance) or referral diagnosis (passive surveillance). *Elevated biomarkers of inflammation* were defined as CRP ≥ 1.1 mg/l or AGP ≥ 0.76 g/l; these levels have been associated with acute infectious disease in this population [28]. Infectious diseases diagnosed by NCMC were categorized as *respiratory, gastrointestinal, malaria* or *other*. Reported signs/symptoms/diagnoses were categorized as: *respiratory:* at least three of: coughing, sneezing, runny nose, sore throat, fever or, diagnosis of respiratory ID from a hospital or clinic; *gastrointestinal:* at least two of: diarrhea, vomiting, stomachache, fever or, diagnosis of gastrointestinal ID from a hospital or clinic; *malaria:* at least two of: fever, headache, weakness/fatigue or, diagnosis of malaria from a hospital or clinic; and, *other:* diagnosis of ID not included in the categories above from a hospital or clinic. Intact CMI was identified by positive DTH-*Candida*, defined as a mean induration diameter ≥ 5 mm.

Potential confounding variables were described as follows: *Age* was calculated from reported date of birth. Weight for height (WHZ) and height for age (HAZ) Z-scores were calculated with EPI INFO software (Centers for Disease Control and Prevention, Atlanta, GA) and wasted (WHZ < −2) and stunted (HAZ < −2) children identified; triceps skinfold (TSF) thickness was considered as a continuous variable. Early weaning (before the recommended age of 2 years) was identified based on caregivers’ report of breastfeeding status and/or weaning age. Sex, housing materials, family size and household animals were characterized based on caregivers’ report.

Data were analyzed with StataMP 13 software (Statacorp; College Station, TX). Calculations of incidence rates (IR) were restricted to the first episode of an infectious disease outcome. Estimates of rates of infectious diseases based on reported signs/symptoms were included only in descriptive analyses. Logistic regression (LR) models were used to assess associations between iron status and prevalent diagnosed infectious disease, elevated inflammation, and DTH-*Candida*; Cox proportional hazards (CPH) models were used to assess associations between iron status and incident diagnosed infectious disease.

We estimated three versions of each model: crude (Model 1), adjusted for confounding variables (Model 2) and fully adjusted (adjusted for all potentially confounding variables; Model 3). In keeping with simulation studies that support a change-in-estimate criterion (a percent change in the magnitude of an association of interest) as the best strategy to identify confounding [32–34], we used a 10% change-in-estimate criterion: adjusted and crude estimates of the associations between IDE (and IDA) and the outcome of interest were compared for each potential confounding variable; a difference >10% was considered confounding. Elevated inflammation, an outcome variable in LR models of the association between iron status and prevalent infectious disease, was considered as a potential confounding variable in LR models of DTH-*Candida* and CPH models of incident infectious disease. To maintain consistency, all LR models of prevalent diagnosed infectious disease (any, malaria, respiratory) included variables that met the criteria for confounding of the association between IDE (or IDA) and any one infectious disease outcome; the same strategy was used for CPH models of incident infectious disease. Fully adjusted models included all potentially confounding variables, with exceptions for variables capturing very similar characteristics: TSF was included, to the exclusion of wasting and stunting; household cattle ownership was included, to the exclusion of household ownership of any animals.

We defined ‘significant’ associations as those with *P* ≤ 0.05; however, we report the magnitude of associations with *P* ≤ 0.20, as these secondary data analyses may be underpowered to detect some associations.

## RESULTS

Three hundred and fourteen children participated in data collection. Complete information on age, sex, infectious disease by reported symptoms and physician’s diagnosis, iron status, DTH*-Candida* and household characteristics was available for 283 participating children; of these, complete information on biomarkers of inflammation was available for 265; complete information on wasting and stunting was available for 267; and, complete information on was TSF available for 239.

Iron deficiency was common among participating children: IDE was identified among 31.1% and IDA among 15.9% (Table 1). NIDA was identified among 14.8% of children. Only 38.2% of children were iron replete.

**Table 1.** Sample characteristics^a^

Iron status		
Replete	108	38.2%
Moderate iron deficiency (iron deficient erythropoiesis, IDE)	88	31.1%
Severe iron deficiency (iron deficiency anemia, IDA)	45	15.9%
Non-iron deficiency anemia (NIDA)	42	14.8%
Prevalent infectious disease		
Biomarkers of inflammation^b^	153	57.7%
Reported symptoms		
Any infectious disease	88	31.1%
Malaria	56	19.8%
Respiratory infectious disease	65	23.0%
Diarrheal/gastrointestinal infectious disease	27	9.5%
Other infectious disease	1	0.4%
Physician’s diagnosis		
Any infectious disease	54	19.0%
Malaria	22	7.8%
Respiratory infectious disease	35	12.4%
Diarrheal/gastrointestinal infectious disease	0	0.0%
Other infectious disease	11	3.9%
Incident infectious disease		
Diagnosis^c^		
Any infectious disease	0.28 per child-month	
Malaria	0.05 per child-month	
Respiratory infectious disease	0.15 per child-month	
Symptoms		
Any infectious disease	0.88 per child-month	
Malaria	0.57 per child-month	
Respiratory infectious disease	0.69 per child-month	
Individual characteristics		
Sex		
Female	154	54.4%
Male	129	45.6%
Age (mean, standard deviation)	4.49, 1.62	
Wasting	1	0.4%
Stunting	75	28.1%
Triceps skin-fold thickness (TSF; mean, SD)	11.78, 3.25	
Weaned ‘early’ (age <2 years)	62	22.8%
Household characteristics		
Materials		
Cement	144	50.9%
Earth	139	49.1%
Animals		
Any	257	91.1%
Cattle	179	63.3%

a Complete information on age, sex, infectious diseases by reported symptoms and physician’s diagnosis, iron status and household characteristics was available for 283 participating children. Of these, complete info on biomarkers of inflammation was available for 265; complete information on wasting and stunting was available for 267; and, complete information on was TSF available for 239.

b Elevated C-reactive protein (CRP) or α_1_-acid glycoprotein [28].

c Few cases of diarrheal/gastrointestinal or other ID were diagnosed, so incidence rates were not calculated.

The prevalence of elevated inflammation and infectious disease was relatively high. Biomarkers of inflammation were elevated among 57.7% of participating children. Signs/symptoms of infectious disease were reported for 31.1% of participating children in the 2-week preceding initial data collection. The prevalence of any clinically apparent infectious disease at the time of initial data collection, by physician’s diagnosis, was 19.0% (malaria, 7.8%; respiratory infectious disease, 12.4% and, other infectious disease, 3.9%).

The incidence of infectious disease was also high: infectious diseases overall were diagnosed at a rate of 0.28 per child-month, or 28 per 100 children per month; malaria was diagnosed at a rate of 5 per 100 children per month; and, respiratory ID were diagnosed at a rate of 15 per 100 children per month. Signs/symptoms of infectious disease were reported at higher rates than diagnoses upon active surveillance of 3–5-year olds.

Results of logistic regression (LR) show generally positive associations between prevalent diagnosed infectious diseases or elevated inflammation and IDA (Table 2; Supplementary Table S1 shows effect estimates for all variables in these models). Control for age, sex, and triceps skinfold thickness (TSF) met the 10% change-in-estimate criterion for confounding (Table 2, Model 2). No additional confounding was apparent by early weaning, home construction materials, or household cattle. IDA was positively associated with any physician-diagnosed infectious disease and elevated inflammation. The crude association between IDA and elevated biomarkers of inflammation (OR: 2.63; *P* = 0.017) lost significance with control for confounding variables (aOR: 2.25, *P* = 0.095 in Model 2; aOR: 2.71; *P* = 0.053 in Model 3). Among physician-diagnosed infectious diseases, this pattern was apparent for diagnosed malaria, but not respiratory infectious disease; associations between IDA and any infectious disease (aOR: 2.42; *P* = 0.091 in Model 2; aOR: 2.90; *P* = 0.047 in Model 3) or malaria (aOR: 3.75; *P* = 0.059 in Model 2; aOR: 4.45; *P* = 0.036 in Model 3) were statistically significant at *P* < 0.05 only when controlling for all potentially confounding variables. NIDA was non-significantly positively associated with any diagnosed infectious disease. Too few cases of additional infectious disease categories were diagnosed to evaluate their associations with iron status using LR. No associations between IDE and infectious disease outcomes were apparent in LR models.

**Table 2.** Logistic regression models evaluating associations between iron status and prevalent infectious disease (reference: iron replete)

	Model 1:			Model 2:			Model 3:		
	Crude^a^			Adjusted for identified confounding variables^b^			Adjusted for all individual characteristics^c^		
	OR	95% CI	*P*-value	aOR	95% CI	*P*-value	aOR	95% CI	*P*-value
Outcome: physician’s diagnosis of any infectious disease									
Iron-deficient erythropoesis	0.69	0.32, 1.51	0.359	0.84	0.34, 2.06	0.698	0.79	0.31, 2.01	0.614
Iron deficiency anemia	1.42	0.62, 3.28	0.407	2.42	0.87, 6.79	0.091	2.90	1.02, 8.30	0.047
Non-iron deficiency anemia	1.56	0.67, 3.62	0.300	1.92	0.75, 4.94	0.174	2.10	0.80, 5.52	0.131
Outcome: physician’s diagnosis of malaria									
Iron-deficient erythropoesis	1.25	0.42, 3.70	0.691	1.37	0.40, 4.74	0.620	1.44	0.41, 5.08	0.568
Iron deficiency anemia	1.80	0.54, 6.02	0.337	3.75	0.95, 14.76	0.059	4.45	1.10, 18.00	0.036
Non-iron deficiency anemia	1.11	0.27, 4.51	0.884	1.41	0.34, 5.92	0.637	1.37	0.32, 5.84	0.666
Outcome: physician’s diagnosis of respiratory infectious disease									
Iron-deficient erythropoesis	0.62	0.25, 1.54	0.302	0.63	0.21, 1.92	0.413	0.51	0.15, 1.70	0.272
Iron deficiency anemia	0.95	0.34, 2.64	0.928	1.58	0.46, 5.43	0.465	1.75	0.50, 6.07	0.379
Non-iron deficiency anemia	1.03	0.37, 2.87	0.950	1.69	0.57, 4.99	0.345	1.87	0.62, 5.67	0.268
Outcome: elevated biomarkers of inflammation^d^									
Iron-deficient erythropoesis	1.00	0.55, 1.79	0.991	0.76	0.39, 1.46	0.408	0.75	0.38, 1.48	0.415
Iron deficiency anemia	2.63	1.19, 5.80	0.017	2.25	0.87, 5.82	0.095	2.71	0.99, 7.41	0.053
Non-iron deficiency anemia	1.57	0.74, 3.31	0.239	1.53	0.67, 3.51	0.310	1.61	0.69, 3.73	0.271

a *N* = 283 for models of physician’s diagnoses; *N* = 265 for model of elevated biomarkers of inflammation.

b Controlling for age, sex and triceps skin-fold thickness; *N* = 239 for models of physician’s diagnoses; *N* = 224 for model of elevated biomarker of inflammation.

c Controlling for age, sex and triceps skin-fold thickness, early weaning, home construction materials, and household cattle ownership; *N* = 233 for models of physician’s diagnoses; *N* = 219 for model of elevated biomarker of inflammation.

d Elevated C-reactive protein (CRP) or α_1_-acid glycoprotein (AGP) [28].

aOR: adjusted odds ratio; OR, odds ratio; CI, confidence interval.

Iron deficient (IDE or IDA) children did not have lower rates of positive responses to the DTH-*Candida* test of CMI; instead, IDA was positively associated with DTH-*Candida* (Table 3; Supplementary Table S2). Age was identified as a confounder in this association. Confounding by sex, anthropometric characteristics, early weaning, home construction material, household cattle, and elevated biomarkers of inflammation was not apparent. The positive relationship between IDA and DTH-*Candida* was significant at *P* < 0.05 only when controlling for age alone (Model 2; OR: 2.34; p: 0.031).

**Table 3.** Logistic regression models evaluating associations between iron status and DTH-*Candida*^a^ (reference: iron replete)

	Model 1: crude^a^			Model 2: adjusted for identified confounding variables^b^			Model 3: adjusted for all individual characteristics^c^		
	OR	95% CI	*P*-value	aOR	95% CI	*P*-value	aOR	95% CI	*P*-value
Outcome: DTH-*Candida*									
Iron deficient erythropoesis	1.14	0.65, 2.00	0.656	1.33	0.74, 2.39	0.340	1.40	0.70, 2.76	0.347
Iron deficiency anemia	1.71	0.84, 3.48	0.139	2.34	1.08, 5.07	0.031	2.60	0.99, 6.85	0.053
Non-iron deficiency anemia	1.14	0.56, 2.33	0.716	1.27	0.61, 2.63	0.516	1.39	0.60, 3.20	0.444

a *N* = 283.

b Controlling for age; *N* = 283.

c Controlling for age, sex, triceps skinfold thickness, elevated biomarkers of inflammation, early weaning, home construction materials, and household cattle ownership; *N* = 219.

aOR, adjusted odds ratio; CI, confidence interval; DTH-*Candida*, delayed-type hypersensitivity to *Candida albicans*; OR, odds ratio.

**Figure 1.**
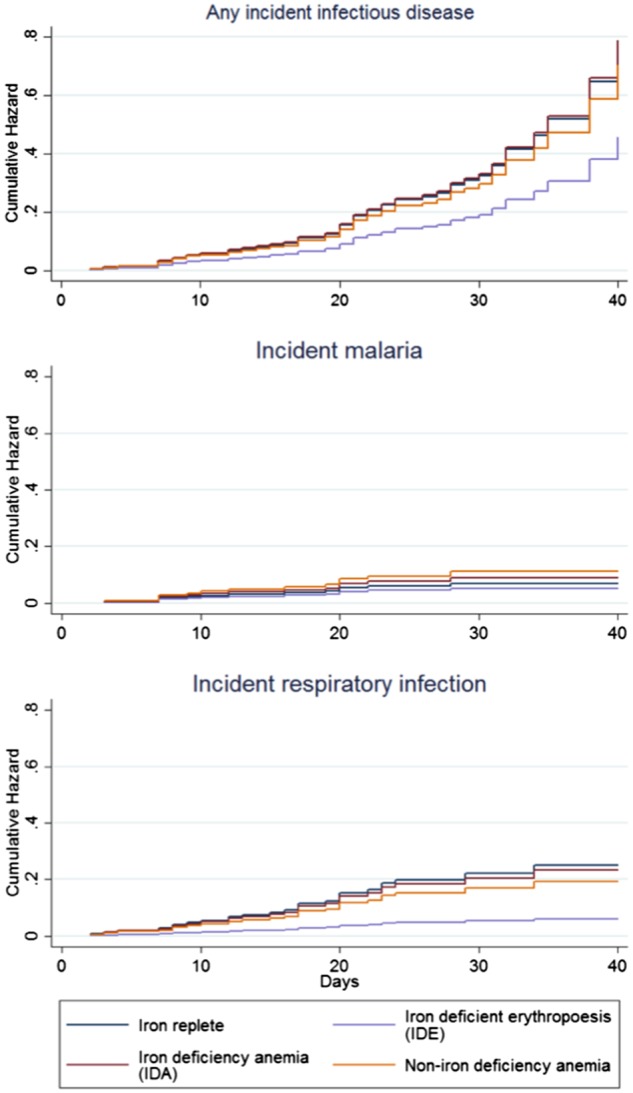
Cox proportional hazard cumulative hazard functions for incident physician-diagnosed infectious disease by iron status. *Replete*: no iron deficiency, no anemia; *iron-deficient erythropoesis* (IDE): iron deficiency, no anemia; *iron deficiency anemia* (IDA): iron deficiency, anemia; *non-iron deficiency anemia* (NIDA): no iron deficiency, anemia

In Cox proportional hazards (CPH) models, the incidence of diagnosed respiratory infectious disease was substantially lower among children with IDE, compared to iron replete (Table 4; Supplementary Table S3). Elevated biomarkers of inflammation and TSF were identified as confounders. Confounding by age, sex, early weaning, home construction materials, or household cattle was not apparent. Risk for respiratory infectious diseases was lower among children with IDE than iron replete (aHR: 0.24; p: 0.030 in Model 2; aHR: 0.25; p: 0.038 in Model 3); a similar, but not significant, association was apparent for infectious disease overall only when controlling for identified confounding variables (aHR: 0.59; p: 0.162 in Model 2). No inverse association was apparent between IDE and malaria in any model. There was no discernable difference in incidence of infectious disease among children with IDA or NIDA compared to iron replete. Cumulative hazard functions are shown in Fig. 1 (estimated from Model 2 in Table 4; Supplementary Table S3).

**Table 4.** Cox proportional hazards models evaluating associations between iron status and incident infectious disease (reference: iron replete)

	Model 1: crude			Model 2: adjusted for identified confounders^a^			Model 3: adjusted for all individual characteristics^b^		
	HR	95% CI	*P*	aHR	95% CI	*P*	aHR	95% CI	*P*
Outcome: physician’s diagnosis of any infectious disease									
Iron-deficient erythropoesis	0.62	0.32, 1.19	0.148	0.59	0.28, 1.24	0.162	0.64	0.31, 1.35	0.243
Iron deficiency anemia	0.95	0.47, 1.92	0.879	1.02	0.45, 2.33	0.962	0.96	0.41, 2.23	0.921
Non-iron deficiency anemia	0.98	0.50, 1.91	0.943	0.91	0.44, 1.87	0.800	1.08	0.53, 2.20	0.841
Outcome: physician’s diagnosis of malaria									
Iron-deficient erythropoesis	0.77	0.18, 3.22	0.718	0.74	0.17, 3.21	0.686	0.75	0.17, 3.36	0.705
Iron deficiency anemia	1.00	0.19, 5.15	0.999	1.28	0.23, 6.87	0.783	1.01	0.18, 5.80	0.991
Non-iron deficiency anemia	1.47	0.35, 6.18	0.595	1.60	0.37, 6.81	0.527	1.65	0.38, 7.22	0.506
Outcome: physician’s diagnosis of respiratory infectious disease									
Iron-deficient erythropoesis	0.31	0.10, 0.93	0.037	0.24	0.07, 0.87	0.030	0.25	0.07, 0.93	0.038
Iron deficiency anemia	1.09	0.44, 2.67	0.854	0.93	0.31, 2.73	0.891	0.79	0.26, 2.38	0.670
Non-iron deficiency anemia	0.87	0.31, 2.40	0.786	0.76	0.25, 2.34	0.639	0.98	0.31, 3.09	0.972

aHR, adjusted hazard ratio; CI, confidence interval; HR, hazard ratio.

a Controlling for triceps skinfold thickness and elevated biomarkers of inflammation.

b Controlling for age, sex, triceps skinfold thickness, elevated biomarkers of inflammation, early weaning, home construction materials and household cattle ownership.

## CONCLUSIONS AND IMPLICATIONS

Consistent with the pattern reported from Tanzanian [19] and in contrast to northern Kenya [6], elevated biomarkers of inflammation and diagnosed infectious diseases were more common, in cross-section, among children with severe iron deficiency (IDA) than iron replete children in Kilimanjaro. However, the *incidence* of diagnosed infectious disease was *lowest* among children with mild-to-moderate IDE; this pattern was restricted to respiratory infectious disease, such that risk for respiratory infection among children with IDE was 76% lower than that of iron replete children. We observed no adverse effect of iron deficiency on CMI (DTH-*Candida*).

We found a protective effect of iron deficiency against respiratory infectious disease, which was limited to IDE; the incidence of respiratory infectious disease was not lower among children with severe iron deficiency (IDA) than among iron replete children. This is consistent with the prediction from Wander *et al.* [6] of a *U*-shaped relationship between iron deficiency and infectious disease risk, produced by the trade-off between the adverse and protective effect of limiting iron availability to both immune cells and infectious agents. However, we found no direct support for the hypothesis that CMI is compromised by iron deficiency; instead, positive DTH-*Candida* (an indicator of intact CMI), was *most* common among children with severe iron deficiency. This finding is not consistent with the trade-off hypothesized by Wander and colleagues [6]. It is possible that the DTH-*Candida* test alone is inadequate to characterize iron deficiency’s impact on CMI; nonetheless, the apparent *U*-shaped relationship between iron deficiency and ID risk remains to be explained.

### Limitations

We must highlight some limitations of these analyses. As secondary analyses of data collected for another purpose, they may be underpowered to detect some hypothesized associations, and risk observing no ‘significant’ association when one truly exists. Parents may have misreported signs/symptoms/diagnoses. Overdiagnosis of malaria, which has been reported in Tanzania and other similar settings [35], may have occurred. Inadequate power and non-differential misclassification of cases may bias our findings toward the null hypothesis. Differential misclassification of malaria cases, wherein overdiagnosis of malaria was more likely among anemic children, is possible; this could create a spurious positive association between anemia (IDA or NIDA) and malaria diagnoses. Thus, we interpret the observed positive cross-sectional association between malaria and IDA with caution.

We observed a protective effect of mild-to-moderate IDE against incident respiratory infectious disease that is consistent with the hypothesis that dietary iron deficiency constitutes a nutritional adaptation to infectious disease. These analyses can provide no information, however, about *how* children became iron deficient. Iron deficiency among children in similar environments arises from both blood loss and destruction due to parasitic infections (e.g. hookworm) [36, 37] *and* dietary iron availability (including low dietary iron intake and high intake of inhibitors of iron absorption) [38, 39]. While we administered anti-helminthic medication to eliminate the effect of helminths on immune outcomes, it is likely that some effect of helminths on iron deficiency and anemia persisted beyond the 1–7 day interval between mebendazole administration and initial data collection.

### Implications

Our most important observation was a protective effect of mild-to-moderate iron deficiency against respiratory infections among young children in Kilimanjaro. In this infectious disease ecology—in which infectious agents are diverse and infectious disease episodes are common [24, 40]—iron deficiency may constitute a *nutritional adaptation* to infectious disease. However, iron deficiency is unlikely to be without cost; growth, motor skills and cognitive development may be compromised among children with IDE. The net impact of iron deficiency on children in Kilimanjaro—in terms of both health and evolutionary fitness—remains to be fully evaluated. Our findings suggest that optimal dietary iron for risk for common infectious diseases falls short of tissue iron needs. If the protection afforded by IDE against infectious diseases outweighs its costs to growth, motor skills, and cognition, even if unhealthy, iron deficiency may constitute a nutritional adaptation in this environment.

In assessing iron deficiency’s possible adaptive value, we must consider not only its impact on risk for common infectious diseases, such as respiratory infections and malaria, but also the relative contribution of these diseases to local child mortality. This is likely to vary across settings. Thus, it is likely that the balance between the benefits of iron deficiency—lower risk for some infectious diseases—and its costs—poorer growth, cognition, and learning—varies for children across infectious disease ecologies. We caution against the broad interpretation that iron deficiency is adaptive, and suggest instead that the adaptive value of iron deficiency should be explicitly evaluated across multiple settings.

### Directions for future research

The heterogeneity of associations between IDE and incident infectious diseases—with IDE inversely associated with respiratory infectious diseases, but not malaria—suggests that testing the hypothesis that iron deficiency represents a nutritional adaptation against infectious disease is best approached with research that neither considers infectious diseases in isolation nor as a single outcome. Research that focuses on one infectious disease, such as malaria, to the exclusion of others, such as respiratory infections, may dangerously mischaracterize the impact of iron nutrition on children’s risk for infectious diseases, while research that considered all infectious diseases as a single outcome may miss the heterogeneous effects of iron nutrition on risk for infection across diverse infectious agents.

It will also be important that future tests of iron deficiency as an adaptation to infectious disease evaluate *how* iron deficiency arises among young children in Kilimanjaro. While we have posited that dietary iron deficiency represents a nutritional adaptation, we are not able to determine how much of the iron deficiency we observed was dietary in nature. Iron deficiency can result from infections (e.g. blood loss during chronic hookworm infection), prolonged inflammation (resulting from infectious or other disease processes), or diets low in bioavailable iron or high in inhibitors of iron absorption. While it is possible that iron deficiency resulting from any of these processes may protect against incident respiratory infections by depriving infectious agents of iron—indeed, depriving infectious agents of iron is the function of the iron-withholding mechanisms of acute inflammation [8]—our hypothesis posits a particular cause of iron deficiency: low dietary iron availability. Future projects should evaluate the proportion of IDE attributable to low dietary intake of iron or high dietary intake of iron inhibitors, as well as the risk for infectious diseases associated with IDE, to more fully address the hypothesis that mild-to-moderate iron deficiency represents a nutritional adaptation to infectious disease.

### Implications for public health interventions

Many authors have urged caution in implementing interventions designed to treat and prevent iron deficiency, such as iron supplementation or fortification, in areas of high malaria transmission, as improvement in iron status is likely to increase children’s risk for severe malaria and death in these settings [15–17, 41]. Similar caution may be warranted in settings with low or no malaria transmission, as our findings suggest that improved iron status may increase children’s risk for respiratory infectious disease in Kilimanjaro. Efforts to improve iron nutrition may be of greatest benefit to East African communities if they are integrated with efforts to improve access to care and prevention, as well as efforts to disrupt infectious disease transmission. Indeed, successful programs to prevent respiratory infections in Kilimanjaro *could* alleviate some dietary iron deficiency without further public health intervention, by changing the infectious disease ecology and the balance between iron deficiency’s costs and benefits.

## Supplementary data

Supplementary data is available at *EMPH* online.

## Supplementary Material

Supplementary Data

Supplementary Tables
