# Supplementary material for: Lower incidence of respiratory infections among iron-deficient children in Kilimanjaro, Tanzania
Source: Evol Med Public Health. 2017 Jun 28;2017(1):109–19. doi: 10.1093/emph/eox010 (PMC5570096; doi:10.1093/emph/eox010)
Supplement: Supplementary Data [file eox010_Supp_1.pdf]

Supplemental Table 1. Logistic regression models evaluating associations between iron status and prevalent infectious disease (reference: iron replete)

| | Model 1: Crude[a] | | | Model 2: Adjusted for identified confounders[b] | | | Model 3: Adjusted for all individual characteristics[c] | | |
|---|---|---|---|---|---|---|---|---|---|
| | OR | 95% CI | P-value | aOR | 95% CI | P-value | aOR | 95% CI | P-value |
| Outcome: physician's diagnosis of any infectious disease | | | | | | | | | |
| Iron deficient erythropoesis | 0.69 | 0.32, 1.51 | 0.359 | 0.84 | 0.34, 2.06 | 0.698 | 0.79 | 0.31, 2.01 | 0.614 |
| Iron deficiency anemia | 1.42 | 0.62, 3.28 | 0.407 | 2.42 | 0.87, 6.79 | 0.091 | 2.90 | 1.02, 8.30 | 0.047 |
| Non-iron deficiency anemia | 1.56 | 0.67, 3.62 | 0.300 | 1.92 | 0.75, 4.94 | 0.174 | 2.10 | 0.80, 5.52 | 0.131 |
| Age | | | | 0.91 | 0.71, 1.17 | 0.455 | 0.90 | 0.69, 1.17 | 0.421 |
| Male sex | | | | 0.68 | 0.34, 1.36 | 0.274 | 0.64 | 0.32, 1.31 | 0.225 |
| Triceps skinfold thickness | | | | 0.84 | 0.73, 0.96 | 0.008 | 0.83 | 0.72, 0.95 | 0.008 |
| Early weaning | | | | | | | 1.79 | 0.79, 4.09 | 0.165 |
| Earth housing material | | | | | | | 1.04 | 0.51, 2.12 | 0.906 |
| Cattle ownership | | | | | | | 1.17 | 0.56, 2.44 | 0.680 |
| Outcome: physician's diagnosis of malaria | | | | | | | | | |
| Iron deficient erythropoesis | 1.25 | 0.42, 3.70 | 0.691 | 1.37 | 0.40, 4.74 | 0.620 | 1.44 | 0.41, 5.08 | 0.568 |
| Iron deficiency anemia | 1.80 | 0.54, 6.02 | 0.337 | 3.75 | 0.95, 14.76 | 0.059 | 4.45 | 1.10, 18.00 | 0.036 |
| Non-iron deficiency anemia | 1.11 | 0.27, 4.51 | 0.884 | 1.41 | 0.34, 5.92 | 0.637 | 1.37 | 0.32, 5.84 | 0.666 |
| Age | | | | 1.09 | 0.77, 1.54 | 0.637 | 1.06 | 0.73, 1.52 | 0.762 |
| Male sex | | | | 0.33 | 0.11, 0.97 | 0.044 | 0.33 | 0.11, 0.98 | 0.045 |
| Triceps skinfold thickness | | | | 0.91 | 0.76, 1.08 | 0.261 | 0.90 | 0.76, 1.07 | 0.249 |
| Early weaning | | | | | | | 0.95 | 0.28, 3.23 | 0.940 |
| Earth housing material | | | | | | | 0.96 | 0.36, 2.55 | 0.942 |
| Cattle ownership | | | | | | | 1.51 | 0.53, 4.33 | 0.439 |
| Outcome: physician's diagnosis of respiratory infectious disease | | | | | | | | | |
| Iron deficient erythropoesis | 0.62 | 0.25, 1.54 | 0.302 | 0.63 | 0.21, 1.92 | 0.413 | 0.51 | 0.15, 1.70 | 0.272 |
| Iron deficiency anemia | 0.95 | 0.34, 2.64 | 0.928 | 1.58 | 0.46, 5.43 | 0.465 | 1.75 | 0.50, 6.07 | 0.379 |
| Non-iron deficiency anemia | 1.03 | 0.37, 2.87 | 0.950 | 1.69 | 0.57, 4.99 | 0.345 | 1.87 | 0.62, 5.67 | 0.268 |
| Age | | | | 0.86 | 0.64, 1.16 | 0.326 | 0.86 | 0.63, 1.17 | 0.338 |
| Male sex | | | | 0.75 | 0.33, 1.70 | 0.487 | 0.67 | 0.29, 1.57 | 0.360 |
| Triceps skinfold thickness | | | | 0.82 | 0.70, 0.96 | 0.016 | 0.81 | 0.68, 0.96 | 0.016 |
| Early weaning | | | | | | | 1.91 | 0.73, 4.98 | 0.188 |
| Earth housing material | | | | | | | 1.24 | 0.53, 2.92 | 0.609 |
| Cattle ownership | | | | | | | 0.72 | 0.30, 1.68 | 0.435 |

| | | | | | | | | | |
|---|---|---|---|---|---|---|---|---|---|
| Outcome: elevated biomarkers of inflammation[d] | | | | | | | | | |
| Iron deficient erythropoesis | 1.00 | 0.55, 1.79 | 0.991 | 0.76 | 0.39, 1.46 | 0.408 | 0.75 | 0.38, 1.48 | 0.415 |
| Iron deficiency anemia | 2.63 | 1.19, 5.80 | 0.017 | 2.25 | 0.87, 5.82 | 0.095 | 2.70 | 0.99, 7.41 | 0.053 |
| Non-iron deficiency anemia | 1.57 | 0.74, 3.31 | 0.239 | 1.53 | 0.67, 3.51 | 0.310 | 1.61 | 0.69, 3.73 | 0.271 |
| Age | | | | 0.87 | 0.71, 1.06 | 0.172 | 0.88 | 0.71, 1.08 | 0.226 |
| Male sex | | | | 0.90 | 0.52, 1.57 | 0.709 | 0.84 | 0.47, 1.49 | 0.546 |
| Triceps skinfold thickness | | | | 0.96 | 0.88, 1.06 | 0.416 | 0.96 | 0.71, 1.08 | 0.226 |
| Early weaning | | | | | | | 1.08 | 0.53, 2.18 | 0.836 |
| Earth housing material | | | | | | | 1.62 | 0.92, 2.88 | 0.097 |
| Cattle ownership | | | | | | | 1.26 | 0.70, 2.27 | 0.449 |

[a] N = 283 for models of physician's diagnoses; N = 265 for model of elevated biomarkers of inflammation
[b] N = 239 for models of physician's diagnoses; N = 224 for model of elevated biomarker of inflammation
[c] N = 233 for models of physician's diagnoses; N = 219 for model of elevated biomarker of inflammation
[d] Elevated C-reactive protein (CRP) or $\alpha_1$-acid glycoprotein [28]
OR: odds ratio; aOR: adjusted odds ratio; CI: confidence interval

Supplemental Table 2. Logistic regression models evaluating associations between iron status and DTH-*Candida*[a] (reference: iron replete)

| | Model 1: Crude[a] | | | Model 2: Adjusted for identified confounding variables[b] | | | Model 3: Adjusted for all potentially confounding variables[c] | | |
|---|---|---|---|---|---|---|---|---|---|
| | OR | 95% CI | P-value | aOR | 95% CI | P-value | aOR | 95% CI | P-value |
| Outcome: DTH-*Candida* | | | | | | | | | |
| Iron deficient erythropoesis | 1.14 | 0.65, 2.00 | 0.656 | 1.33 | 0.74, 2.39 | 0.340 | 1.40 | 0.70, 2.76 | 0.347 |
| Iron deficiency anemia | 1.71 | 0.84, 3.48 | 0.139 | 2.34 | 1.08, 5.07 | 0.031 | 2.60 | 0.99, 6.85 | 0.053 |
| Non-iron deficiency anemia | 1.14 | 0.56, 2.33 | 0.716 | 1.27 | 0.61, 2.63 | 0.516 | 1.39 | 0.60, 3.20 | 0.444 |
| Age | | | | 1.20 | 1.02, 1.40 | 0.027 | 1.35 | 1.09, 1.68 | 0.006 |
| Male sex | | | | | | | 1.12 | 0.63, 1.99 | 0.696 |
| Triceps skinfold thickness | | | | | | | 1.08 | 0.98, 1.19 | 0.129 |
| Elevated biomarkers of inflammation[d] | | | | | | | 0.77 | 0.43, 1.36 | 0.365 |
| Early weaning | | | | | | | 1.11 | 0.55, 2.24 | 0.776 |
| Earth housing material | | | | | | | 0.74 | 0.42, 1.31 | 0.309 |
| Cattle ownership | | | | | | | 0.88 | 0.49, 1.31 | 0.671 |

[a] N = 283
[b] N = 283
[c] N = 219
[d] Elevated C-reactive protein (CRP) or $\alpha_1$-acid glycoprotein (AGP) [28]
DTH-*Candida*: Delayed-type hypersensitivity to *Candida albicans*; OR: odds ratio; aOR: adjusted odds ratio; CI: confidence interval

Supplemental Table 3. Cox proportional hazards models evaluating associations between iron status and incident infectious disease (reference: iron replete)

| | Model 1: Crude | | | Model 2: Adjusted for identified confounders | | | Model 3: Adjusted for all individual characteristics | | |
|---|---|---|---|---|---|---|---|---|---|
| | HR | 95% CI | P | aHR | 95% CI | P | aHR | 95% CI | P |
| Outcome: physician's diagnosis of any infectious disease | | | | | | | | | |
| Iron deficient erythropoesis | 0.62 | 0.32, 1.19 | 0.148 | 0.59 | 0.28, 1.24 | 0.162 | 0.60 | 0.28, 1.29 | 0.191 |
| Iron deficiency anemia | 0.95 | 0.47, 1.92 | 0.879 | 1.02 | 0.45, 2.33 | 0.962 | 0.94 | 0.40, 2.21 | 0.889 |
| Non-iron deficiency anemia | 0.98 | 0.50, 1.91 | 0.943 | 0.91 | 0.44, 1.87 | 0.800 | 0.96 | 0.46, 2.02 | 0.981 |
| Age | | | | | | | 0.84 | 0.68, 1.03 | 0.095 |
| Male sex | | | | | | | 1.06 | 0.60, 1.87 | 0.846 |
| Triceps skinfold thickness | | | | 1.00 | 0.91, 1.09 | 0.925 | 0.96 | 0.86, 1.06 | 0.382 |
| Elevated biomarkers of inflammation[a] | | | | 1.10 | 0.63, 1.92 | 0.730 | 1.12 | 0.63, 1.99 | 0.707 |
| Early weaning | | | | | | | 0.96 | 0.48, 1.93 | 0.911 |
| Earth housing material | | | | | | | 0.66 | 0.37, 1.17 | 0.153 |
| Cattle ownership | | | | | | | 0.98 | 0.54, 1.80 | 0.960 |
| Outcome: physician's diagnosis of malaria | | | | | | | | | |
| Iron deficient erythropoesis | 0.77 | 0.18, 3.22 | 0.718 | 0.74 | 0.17, 3.21 | 0.686 | 0.67 | 0.15, 2.99 | 0.598 |
| Iron deficiency anemia | 1.00 | 0.19, 5.15 | 0.999 | 1.27 | 0.23, 6.87 | 0.783 | 1.04 | 0.18, 6.10 | 0.959 |
| Non-iron deficiency anemia | 1.47 | 0.35, 6.18 | 0.595 | 1.60 | 0.37, 6.81 | 0.527 | 1.62 | 0.37, 7.10 | 0.523 |
| Age | | | | | | | 0.84 | 0.55, 1.28 | 0.410 |
| Male sex | | | | | | | 1.10 | 0.35, 3.51 | 0.870 |
| Triceps skinfold thickness | | | | 1.06 | 0.89, 1.25 | 0.519 | 1.04 | 0.85, 1.26 | 0.716 |
| Elevated biomarkers of inflammation[a] | | | | 0.62 | 0.20, 1.87 | 0.394 | 0.63 | 0.20, 1.95 | 0.420 |
| Early weaning | | | | | | | 1.25 | 0.33, 4.80 | 0.742 |
| Earth housing material | | | | | | | 0.77 | 0.24, 2.41 | 0.648 |
| Cattle ownership | | | | | | | 0.69 | 0.22, 2.19 | 0.533 |
| Outcome: physician's diagnosis of respiratory infectious disease | | | | | | | | | |
| Iron deficient erythropoesis | 0.31 | 0.10, 0.93 | 0.037 | 0.24 | 0.07, 0.87 | 0.030 | 0.23 | 0.06, 0.84 | 0.027 |
| Iron deficiency anemia | 1.09 | 0.44, 2.67 | 0.854 | 0.93 | 0.31, 2.73 | 0.891 | 0.78 | 0.26, 2.40 | 0.668 |
| Non-iron deficiency anemia | 0.87 | 0.31, 2.40 | 0.786 | 0.76 | 0.25, 2.34 | 0.639 | 0.91 | 0.29, 2.89 | 0.876 |
| Age | | | | | | | 0.71 | 0.52, 0.97 | 0.029 |
| Male sex | | | | | | | 1.31 | 0.57, 3.02 | 0.526 |
| Triceps skinfold thickness | | | | 1.05 | 0.93, 1.19 | 0.442 | 1.00 | 0.87, 1.16 | 0.965 |
| Elevated biomarkers of inflammation[a] | | | | 0.96 | 0.44, 2.12 | 0.921 | 0.82 | 0.36, 1.87 | 0.635 |

| | | | |
|---|---|---|---|
| Early weaning | 1.03 | 0.40, 2.68 | 0.951 |
| Earth housing material | 0.64 | 0.27, 1.47 | 0.289 |
| Cattle ownership | 0.89 | 0.38, 2.08 | 0.790 |

[a] Elevated C-reactive protein (CRP) or $\alpha_1$-acid glycoprotein (AGP) [28]
HR: hazard ratio; aHR: adjusted hazard ratio; CI: confidence interval
